# Community-Based Outbreak of *Neisseria meningitidis* Serogroup C Infection in Men who Have Sex with Men, New York City, New York, USA, 2010−2013

**DOI:** 10.3201/eid2108.141837

**Published:** 2015-08

**Authors:** Molly M. Kratz, Don Weiss, Alison Ridpath, Jane R. Zucker, Anita Geevarughese, Jennifer Rakeman, Jay K. Varma

**Affiliations:** New York City Department of Health and Mental Hygiene, New York, New York, USA (M.M. Kratz, D. Weiss, A. Ridpath, J.R. Zucker, A. Geevarughese, J. Rakeman, J.K. Varma);; Centers for Disease Control and Prevention, Atlanta, USA (A. Ridpath, J.R. Zucker, J.K. Varma)

**Keywords:** Neisseria meningitidis, bacteria, disease outbreaks, meningococcal meningitis, invasive meningococcal disease, serogroup C, men who have sex with men, sexual behavior, community networks, internet, New York, New York

## Abstract

In September 2012, the New York City Department of Health and Mental Hygiene identified an outbreak of *Neisseria meningitidis* serogroup C invasive meningococcal disease among men who have sex with men (MSM). Twenty-two case-patients and 7 deaths were identified during August 2010−February 2013. During this period, 7 cases in non-MSM were diagnosed. The slow-moving outbreak was linked to the use of websites and mobile phone applications that connect men with male sexual partners, which complicated the epidemiologic investigation and prevention efforts. We describe the outbreak and steps taken to interrupt transmission, including an innovative and wide-ranging outreach campaign that involved direct, internet-based, and media-based communications; free vaccination events; and engagement of community and government partners. We conclude by discussing the challenges of managing an outbreak affecting a discrete community of MSM and the benefits of using social networking technology to reach this at-risk population.

## Abstract 2

Questions about how to protect this at-risk population deserve careful consideration.

Invasive meningococcal disease (IMD) is a severe infection of the bloodstream and meninges caused by the bacterium *Neisseria meningitidis*. Although most infected persons recover, 10%–15% of cases are fatal, often within 24 hours of symptom onset. An additional 11%−19% survive with serious neurologic or other complications (*1*). *N. meningitidis* colonizes the nasopharynx and is transmitted through close or prolonged contact. Functional asplenia, complement deficiency, and infection with HIV increase sporadic IMD risk, and living in close quarters, smoking, attending bars, and kissing have been associated with IMD outbreaks (*2**,**3*).

In the United States, IMD occurs rarely and has been decreasing over the past 25 years; the estimated incidence rate for illness caused by all *N. meningitidis* serogroups in 2012 was 0.15 cases/100,000 persons, and only 0.03 cases/100,000 persons for serogroup C meningococcal disease (*4**,**5*). Infants and young children are the most at risk, followed by persons >65 years of age. Outbreaks, which account for only 2% of reported cases, generally involve small numbers of cases and occur in both community and institutional settings (*3*). Only 2 IMD outbreaks, both serogroup C meningococcal disease, have been reported as occurring exclusively among MSM; 1 comprised 6 cases in Toronto, Ontario, Canada, in 2001, and another 6 cases in Chicago, Illinois, USA, in 2003. Both outbreaks prompted vaccination campaigns targeting MSM; 3,850 were vaccinated in Toronto and 14,267 in Chicago (*6**,**7*).

The first outbreak of IMD in New York City, New York, in >25 years occurred during 2005−2006 and included 23 cases of serogroup C meningococcal disease among current and former illicit drug users and their contacts. The outbreak occurred in a contiguous 4-ZIP code area of central Brooklyn and was resolved after 2,763 persons were vaccinated in a targeted campaign (*8*). In 2012, the New York City Department of Health and Mental Hygiene (DOHMH) identified the third North America outbreak of serogroup C meningococcal disease occurring among MSM (a fourth outbreak was recognized in Chicago, Illinois, USA in May 2015 [http://www.bcbsil.com/pdf/education/cdph_press_release.pdf]). We report the epidemiology of the 2012 New York City outbreak and efforts of DOHMH to control it.

## Methods

### Surveillance and Case Investigation

The New York City Health Code requires that IMD cases be reported immediately to DOHMH. Every reported IMD case is investigated to confirm the diagnosis, identify close contacts for antimicrobial drug prophylaxis, obtain risk factor information, characterize the isolate by serogroup and by pulsed-field gel electrophoresis (PFGE) at the New York City Public Health Laboratory. Case investigations include medical record review and interviews with health care providers, family members, close contacts, and the patient when possible. Antimicrobial drug prophylaxis is offered to persons who had prolonged close contact, both sexual and non-sexual, with the patient during the infectious period. For this outbreak, the case definition used was a clinically compatible illness for serogroup C meningococcal disease meeting the 2010 Council of State and Territorial Epidemiologists case definition for a confirmed or probable case in an MSM (*9*).

After DOHMH identified the serogroup C meningococcal disease outbreak and its association with MSM in September 2012, case investigators attempted to re-interview all male IMD case-patients in New York City since 2010 to explicitly inquire about sexual identity and behavioral risk factors and to identify any additional cases. Although sexual contacts are routinely elicited during all IMD case investigations, DOHMH staff did not previously record information about a patient’s sexual identity or sexual history beyond their period of infectiousness. During the outbreak period, 7 cases of serogroup C meningococcal disease were diagnosed in non-MSM; these cases were not considered to be part of the outbreak.

### Vaccine Initiative and Tracking Vaccine Uptake

After the vaccine recommendation was made, DOHMH implemented an immunization initiative consisting of vaccine distribution and outreach events. DOHMH began tracking the aggregate number of doses administered at its clinics. In addition, public and private health care facilities with large MSM and HIV-infected patient populations were asked to make regular summary reports of meningococcal vaccine administration to MSM. The size of the population targeted for vaccination was estimated by using data from the New York City Community Health Survey and the HIV Surveillance Registry of DOHMH.

In the United States, quadrivalent meningococcal vaccine is used routinely and provides protection against *N. meningitidis* serogroups A, C, W, and Y. One dose is recommended for children 11–12 years of age, with a booster dose at age 16. This vaccine is also routinely recommended for first-year college students living in residential housing, travelers to disease-endemic areas, laboratory workers, and those at increased clinical risk (*10*).

## Results

### Outbreak

**Figure 1:**
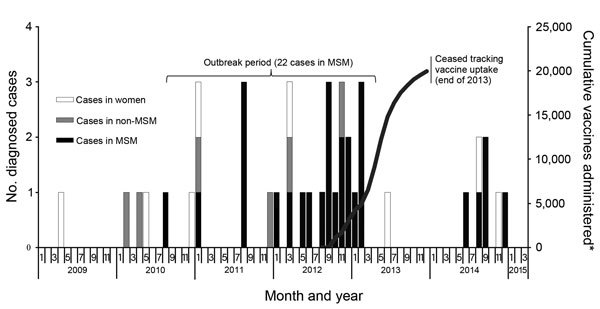
Monthly invasive serogroup C incidence and cumulative vaccine uptake, New York City, New York, USA, 2009–2015. *Vaccine uptake among MSM only as part of outbreak response. MSM, men who have sex with men; non-MSM, men who do not have sex with men.

We identified 22 outbreak-related cases of serogroup C meningococcal disease during August 2010−February 2013 (Figure 1). The first case occurred in August 2010, and 8 additional cases occurred intermittently over the next 24 months. Transmission increased in September 2012; a cluster of 4 cases was diagnosed within 4 weeks. All 4 case-patients were HIV infected, and 2 reported sexual contact with each other. At that time, DOHMH formally recognized the outbreak, issued a vaccine recommendation (on October 4, 2012), and began implementing response efforts. The initial provider recommendation was to administer meningococcal vaccine to “HIV-infected men who are New York City residents and who had intimate contact with a man met either through an online website, digital application (app), or at a bar or party since September 1, 2012” (*11*). In accordance with the official Centers for Disease Control and Prevention (CDC) recommendation, those who were HIV infected and considered themselves at risk were encouraged to get a second dose of vaccine 8 weeks after the first dose (*10*).

Three additional cases of serogroup C meningococcal disease (1 in an HIV-infected person) were identified in MSM before mid-November 2012, shifting the epidemiology of the outbreak to indicate an association with certain neighborhoods in Brooklyn. This finding prompted revision of the vaccine recommendation to “men who have sex with men, regardless of HIV status, if they live in specific areas of Brooklyn and report intimate contact with a man met either through an online website, digital application (app), or at a bar or party since September 1, 2012” (*12*). Six additional cases were detected (3 in HIV-infected persons), including a cluster of 4 in early 2013, but the association with Brooklyn weakened. DOHMH further expanded the target population for vaccination in March 2013 to include all areas of the city and all HIV-infected MSM, regardless of risk behavior, in addition to MSM not infected with HIV who met the risk criteria (*13*). With no new cases identified 12 months after the final February 2013 case, DOHMH declared the outbreak over in March 2014.

Of the 22 outbreak-related cases identified, 7 (32%) were fatal. Co-infection with HIV was present in 12 (55%) case-patients, 5 (42%) of whom died. The age range of the case-patients was 21−59 years (median 31 years, mean 34 years). Case-patients lived throughout the city; the largest proportion (10 [45%]) lived in Brooklyn. Eleven (50%) case-patients were African American, and 4 (18%) were Hispanic or other. Eleven (50%) reported using recreational drugs; of the 15 case-patients for which a determination could be made, 9, including the index case-patient; reported using websites and mobile phone applications (apps) to connect with other men looking for sexual partners (Table 1).

**Table 1:** Epidemiology and risk factors of for 22 outbreak case-patients with invasive meningococcal disease, New York City, New York, USA, 2010–2013*

Characteristic	Value	%
Age, y		
Range	21–59	NA
Mean	34	NA
Median	32	NA
Borough†		
Brooklyn	10	45
Manhattan	7	32
Bronx	2	9
Queens	2	9
Race/ethnicity		
African American	11	50
Caucasian	6	27
Hispanic or other	4	18
Asian	1	5
Risk behavior		
Using recreational drugs	9	41
Using websites or phone apps to meet sexual partners‡	9	60
HIV infected	12	55
Outcome		
Died	7	32
Died, HIV infected	5	42

*NA, not applicable. †One case-patient was reported being homeless. ‡A determination could be made for only 15 of the 22 outbreak case-patients.

Isolates from 15 case-patients were available for PFGE; 14 (93%) isolates were >85% related to the unique outbreak strain, and 9 (64%) of these were indistinguishable from it. These 15 case-patients had culture-confirmed results. The remaining 7 case-patients had culture-negative results; these 7 case-patients were given a diagnosis by PCR. During the outbreak period, an additional 7 cases were diagnosed in non-MSM; isolates from 6 were available for PFGE and only 2, both in women, were found to be >85% related to the outbreak strain. None of the cases in non-MSM were considered to be part of the outbreak.

DOHMH estimated that in 2012, MSM in New York City were 52.5 times (95% CI 19.9−137.4) more likely to contract IMD than other male residents 18−64 years of age (12.6 cases/100,000 MSM compared with 0.24 cases/100,000 unknown or non-MSM). Furthermore, HIV-infected MSM (21.2/100,000) had 88.3 times (95% CI 30.5−252.9) the relative risk for infection than unknown or non-MSM residents of New York City in 2012 (Table 2).

**Table 2:** Rates for invasive meningococcal disease for residents (all serogroups) of New York City, New York, USA, 2012*

Category	No. cases	Population at risk	Rate/100,000	Relative risk	95% CI
All New York City	25	8,175,133	0.31	NA	NA
Men	21	3,882,544	0.54	NA	NA
Women	4	4,292,589	0.09	NA	NA
All New York City, age 18–64 y	21	5,413,864	0.39	NA	NA
Women	2	2,827,956	0.07	NA	NA
Men	19	2,585,908	0.73	NA	NA
Men, not/unknown MSM	6	2,482,908	0.24	Referent	NA
MSM	13	103,000	12.6	52.5	19.9–137.4
MSM with HIV/AIDS	8	37,720	21.2	88.3	30.5–252.9
MSM without HIV/AIDS	5	65,280	7.7	32.1	9.7–103.9

*NA, not applicable; MSM, men who have sex with men.

### Vaccination Initiative

After the initial recommendation was issued, vaccine was made available, free of charge, at DOHMH clinics in 9 locations throughout New York City. DOHMH staff also identified private health care facilities with large HIV-infected and MSM patient populations and encouraged them to administer vaccine to persons at risk and facilitated procurement and distribution of vaccine when necessary. It is estimated that >20,000 men were vaccinated with >1 dose during October 2012−August 2013 in New York City.

DOHMH also staged free vaccine events at bars and clubs geared toward MSM to provide convenient, stigma-free opportunities for men at risk to get vaccinated. A prominent MSM party promoter was hired to host and oversee marketing of these events, including the creation of a Facebook page and a promotional YouTube video (*14*). Vaccine uptake was much lower than expected; health officials administered only 85 doses at 4 DOHMH-sponsored events. Recognizing the value of supporting established patient–provider relationships, DOHMH also supplied vaccine for events hosted by clinicians with a history of treating HIV-infected and MSM patients. These events were staged on-site at their clinical facilities, as well as off-site at MSM bars, sex clubs, and 2013 NYC Pride parades and parties. Demand was much higher at these non-DOHMH events, which facilitated the administration of >2,800 doses of vaccine.

### Outreach Campaign

To promote vaccination among the at-risk subset of the MSM population in New York City, DOHMH began a multifaceted outreach campaign in October 2012. The components of the campaign can be divided into 2 general categories.

#### Direct and Internet-Based Outreach

**Figure 2:**
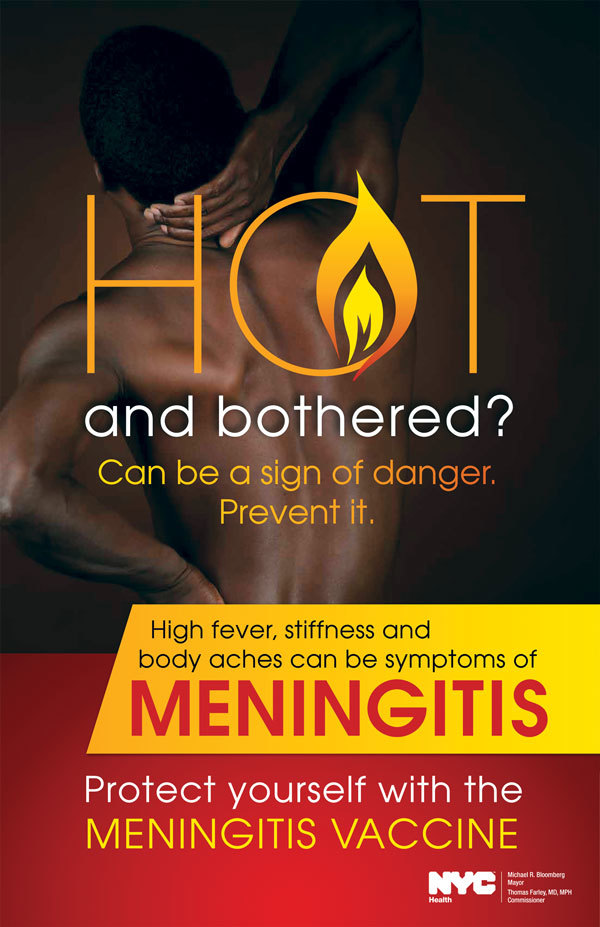
Meningococcal vaccination campaign outreach poster, New York City, New York, USA.

DOHMH provided updates to health care providers through the Health Alert Network, mailed and posted educational materials, and resources to facilitate vaccination (e.g., screening forms, consent forms, billing information) on its website and gave presentations at academic medical centers, large clinics, and community-based organizations. To reach MSM at risk, DOHMH distributed >1,200 posters (Figure 2) and >70,000 palm cards at locations geared toward MSM through established community-based partners participating in the DOHMH condom distribution program. Materials were printed in English, Spanish, Chinese, Japanese, Korean, and Thai.

Beginning in October 2012, DOHMH disseminated information through Twitter and >100 websites and blogs with high MSM viewership and paid for banner and pop-up advertisements on websites and apps targeting MSM, including the app Grindr. In addition, email messages were sent en masse to all registered members of Manhunt, DaddyHunt, and Adam4Adam. When possible, communications were targeted to reach only users in New York City and, before the expansion of the vaccine recommendation, only users who identified themselves as HIV infected.

Click-through proportions, defined as the number of persons who saw the advertisement/ message and clicked to get more information divided by the number of times the advertisement/message appeared, were used to evaluate the effect of these communications. In November 2012, a total of 40,116 (0.82%) of 488,000 pop-ups ads and 2,782 (0.06%) of 463,645 banner ads were clicked on, compared with 87 (14.5%) of 605 email blasts to users of a popular hook-up website. Of 266 users surveyed, 118 (44%) recalled receiving an email about the outbreak and 77 (29%) of users recalled seeing one of the banner ads run on the site.

#### Engagement of External Partners and Mainstream Media Coverage

To promote vaccination, DOHMH communicated with and distributed educational materials to community organizations and elected officials representing large numbers of MSM. To address challenges with provider reimbursement, New York state regulators notified health insurers that all insurance plans must cover the cost of meningococcal vaccination. DOHMH also worked with legislators to help design a bill authorizing New York pharmacists to administer vaccine; this bill was signed into law by the governor in July 2013.

**Figure 3:**
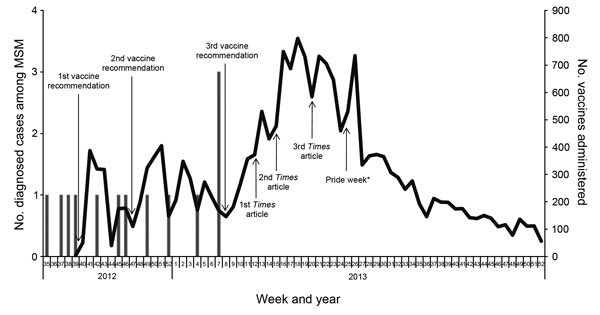
Weekly invasive meningococcal disease incidence among men who have sex with men (MSM) and vaccine uptake, New York City, New York, USA, August 2012–2013, encompassing the last of 13 of 22 outbreak cases. The Department of Health and Mental Hygiene ceased tracking of vaccine uptake at the end of 2013. Dates of article in *The New York Times* are indicated. *Free vaccinations were provided at many of the parades and events during NYC Pride.

Some small-scale print and electronic publications reported the story during the first few months of the vaccine campaign, but it was not until *The New York Times* ran a comprehensive article on March 21, 2013, more than a month after the last associated case was diagnosed, that the outbreak was featured in a variety of other more high-profile publications and engendered substantial coverage on social media sites (*15*). This pattern was repeated with 2 subsequent articles in *The New York Times* that provided updates on the outbreak; both articles also spurred additional reporting by other media outlets (*16**,**17*). These articles appeared to have a much more substantial effect on vaccine uptake than any of the other outreach initiatives (Figure 3).

## Discussion

This protracted IMD outbreak posed several administrative challenges and inspired innovative management and outreach strategies that could benefit responses to other public health emergencies. This outbreak was notable for its association with men using websites and apps to find male sex partners.

Communicable diseases spread through social networks, but because internet and mobile technologies facilitate spontaneous, often anonymous sexual encounters, they complicate understanding of these networks and identification of contacts. We were not able to link cases directly to each other, a single website or app, or a venue, hindering efforts to clearly and narrowly define the target population. Nine (60%) of the 15 cases for which case investigators could make a determination reported the risk behavior of using hook-up websites and apps to find sexual contacts; the true proportion may well be higher, considering the sensitive nature of the inquiry.

It was difficult to decide whether and when to implement a vaccination campaign in the context of a slow moving outbreak. By the end of September 2012, we had identified 13 outbreak-related cases spread out over >2 years; 4 of these cases had been diagnosed in the previous 3 months. CDC defines a community-based outbreak as having a primary attack rate of >10 cases/100,000 persons within a 3-month time frame (*18*). Using results from the New York City Community Health Survey of the DOHMH, we estimated the 2012 population of MSM in New York City to be ≈103,000 and calculated a serogroup C meningococcal disease attack rate of 3.9 cases/100,000 persons (July−September 2012), which is well below the CDC recommended threshold for mass vaccination. Although we were unable to make a precise determination, we proposed that the actual number of MSM at risk was a relatively small subset of the total MSM population. Thus, the true attack rate of the outbreak was probably much higher than 3.9 cases/100,000 persons. Furthermore, we considered the duration and severity of the outbreak, including the highly increased relative risk for MSM of contracting serogroup C meningococcal disease compared with that for non-MSM in New York City (Table 2) when concluding that a citywide vaccination campaign was warranted.

In constructing messages to the target population, the language had to be broad enough to reach the discrete person at risk while also being specific enough to appeal to them, and these messages were criticized for what some perceived to be a vague recommendation centered on an already stigmatized population. As the outbreak evolved, we issued 2 revisions to our initial vaccine recommendation with the intent of better capturing the MSM subset population at highest risk. However, providers and the public alike had difficulty digesting the changing message while combating fatigue and apathy toward the outbreak response efforts.

To reach those persons eligible for vaccination, we expanded on a traditional outreach approach to include more innovative, internet-based methods. When we considered click-through rates and the average cost per click, email blasts to members of hook-up websites proved a successful, comparatively cost-effective method for messaging the at-risk population. However, electronic outreach supplemented but did not substitute for traditional media. We recorded major spikes in vaccine demand after articles about the outbreak were published in *The New York Times*, a widely read publication, and uptake continued to increase as smaller-scale print and electronic publications and social media participants picked up the story. This response suggests that, when used in tandem, traditional and social media can exponentially increase the effect of outreach. Traditional media can be used to penetrate mainstream society with a specific message, and social media can be used to expand and target that message to hard-to-reach populations. As the popularity of social media continues to increase, efforts to control future outbreaks of infectious disease should adapt to using technologies that are flexible, far-reaching, and cost-effective.

Our multifaceted response and outreach efforts to control the serogroup C meningococcal disease outbreak among MSM in New York City increased awareness and were associated with increased vaccine administration, which peaked after the diagnosis of the final case in February 2013. However, in June 2014, fifteen months after the last outbreak-related case was identified, another case of serogroup C meningococcal disease in MSM in New York City was reported to DOHMH (Figure 1). This case was followed by a cluster of 3 cases in late August and early September. These 3 case-patients were determined to be part of the same social network and to have had contact with each other before their onset dates of serogroup C meningococcal disease. DOHMH launched another round of outreach efforts, including ads on hook-up sites and apps, and held 3 free vaccination events, and *The New York Times* published an article covering the cluster (*19*). One additional case of serogroup C meningococcal disease in MSM was diagnosed in December 2014. All 5 of these post-outbreak case-patients were HIV infected, and PFGE testing showed the infecting strain to be closely related to the outbreak strain circulating in 2012. Two of the 5 case-patients had been vaccinated (compared with only 1 of the 14 outbreak case-patients with known vaccination status), and all survived their infections.

As of the publication of this article, the DOHMH recommendation to vaccinate all HIV-infected MSM (with 2 doses) and HIV-uninfected MSM engaging in risk behavior, first issued in March 2013, remains in effect. Since 2010, a total of 27 MSM case-patients with serogroup C meningococcal disease have been identified in New York City and 17 (63%) of them were HIV infected. Los Angeles, California; France; and Germany have also reported clusters of MSM case-patients with serogroup C meningococcal disease, many HIV infected, prompting their own vaccine recommendations (*20**−**22*). However, the behavioral and microbiologic factors leading to transmission of serogroup C meningococcal disease among MSM are still not understood and require attention as additional epidemiologic data are collected and analyzed. In addition, questions about how to protect this at-risk population deserve careful consideration, including whether national guidelines should recommend routine meningococcal vaccination of HIV-infected MSM.
